# Cross-species comparison in nonclinical pharmacokinetics of lenvatinib by a simple HPLC with ultraviolet detection

**DOI:** 10.1038/s41598-023-35297-z

**Published:** 2023-05-23

**Authors:** Hitoshi Mizuo, Yuji Mano

**Affiliations:** 1 Global Drug Metabolism and Pharmacokinetics, Eisai Co., Ltd., Tokodai 5-1-3, Tsukuba, Ibaraki 300-2635 Japan; 2 Laboratory of Genomics-Based Drug Discovery, Faculty of Medicine, Graduate School of Comprehensive Human Sciences, University of Tsukuba, Tennodai 1-1-1, Tsukuba, Ibaraki 305-8575 Japan

**Keywords:** Drug discovery, Biological techniques

## Abstract

Lenvatinib (Lenvima) is a tyrosine kinase inhibitor on the market and has been used for the treatment of various types of cancer. It is important to understand differences in pharmacokinetics (PK) between nonclinical animals and humans, and thus, we evaluated PK of lenvatinib in mice, rats, dogs, and monkeys. A simple assay for lenvatinib was developed by high performance liquid chromatography with ultraviolet detection and validated in accordance with the bioanalytical guidelines. Lenvatinib was quantifiable at 5–100,000 ng/mL using 50 μL of plasma. Accuracy and precision in the intra- and inter-batch reproducibility were within the acceptance criteria, indicating a robust assay. Lenvatinib was intravenously or orally administered to mice, rats, dogs, and monkeys to fully characterize the cross-species PK. Total clearance and volume of distribution were relatively low and bioavailability of lenvatinib was approximately 64–78% in all the species tested. PK of lenvatinib in mice and rats after oral dose was almost linear at the doses ranging from 3 to 30 mg/kg. An empirical allometric scaling successfully predicted oral systemic exposure of lenvatinib in humans. Collectively, PK profiles of lenvatinib in nonclinical animals were well characterized and were useful for PK prediction in humans.

## Introduction

Lenvatinib (LENVIMA^®^) is a novel, multi-targeted, tyrosine kinase inhibitor that inhibits vascular endothelial growth factor receptors 1–3, fibroblast growth factor receptors 1–4, platelet-derived growth factor receptor α, c-Kit and RET^1–3^. Lenvatinib showed anti-tumor activities against a variety of human cancer cell lines in mouse xenograft models^1,2^, but its pharmacokinetics (PK) remains to be reported. To fully understand the pharmacological activities as well as potential toxicological profiles of lenvatinib, it is important to evaluate PK profiles, and thus, in the present study, we evaluated PK of lenvatinib in four animal species including mice, rats, dogs, and monkeys. To characterize PK profiles of drugs, it is critical to establish bioanalytical methods with sufficient sensitivity and reproducibility. In addition, it is also important to develop assays by less expensive assay platforms which can be available for most laboratories. The assay methods of lenvatinib have been reported mostly by liquid chromatography with tandem mass spectrometry (LC–MS/MS)^4,5^, however, publications on assays of lenvatinib by high-performance liquid chromatography (HPLC) with ultraviolet detection are very limited despite that it is a simple and inexpensive assay platform. Assays by a simple HPLC often encounter selectivity issues, however, selectivity issues can be overcome by optimization in the extraction procedure even in the HPLC assays. The dynamic range for quantification is often an issue in the LC–MS/MS assay, however, it is often the case that wider dynamic ranges can be achieved in the HPLC assays, which is advantageous in assessments of PK profiles in experimental animals where higher drug concentrations are expected due to higher drug doses than those in humans. Assays with wide dynamic ranges decrease the chances of re-assay of samples whose concentrations are above the upper limit of quantification (ULOQ), which increases assay throughput. Indeed, the highest dose tested in the oral dose PK study of lenvatinib in animals was 30 mg/kg, which was much higher than the clinical dose of lenvatinib up to 24 mg/subject (ca. 0.34 mg/kg assuming the body weight of 70 kg), and plasma lenvatinib levels in animals were much higher than those in humans. The assay developed in this study achieved a 20,000-fold dynamic range from 5 ng/mL as the lower limit of quantification (LLOQ) to 100 μg/mL by the HPLC with ultraviolet detection (HPLC–UV). To the best knowledge of the authors, this study is the first report for the assay of lenvatinib in cross-species animals by HPLC–UV, and the assay can be applicable across animals given that careful optimization enables the assay to be applied to the four animal species including mice, rats, dogs, and monkeys. Characterization of PK of drugs in experimental animals is important to understand species differences in pharmacological and toxicological findings, and it is also critical to predict PK of drugs in humans using PK data in the experimental animals. Accurate prediction of PK in humans enables us to design the first-in-man clinical trial appropriately with suitable design of active pharmaceutical ingredient for pharmaceutical formulation. Allometric scaling of PK parameters from those in animals to humans is a typical empirical approach for predicting human PK and studies confirmed its usefulness in human PK prediction^6^. In the present study, we predicted PK parameters of lenvatinib in humans using the allometry scaling approach with PK data in mice, rats, dogs, and monkeys, then compared the predicted data to the observed one in humans.

## Methods

### Chemicals and reagents

Lenvatinib and ER-227326 [internal standard (IS)] were synthesized at Eisai Co., Ltd. (Ibaraki, Japan). Drug-free blank plasma containing sodium heparin as an anticoagulant from mice and rats was obtained from Charles River Japan Inc. (Kanagawa, Japan), and that from dogs and monkeys was obtained from Eisai Co., Ltd. (Ibaraki, Japan) and purchased from Shin Nippon Biomedical Laboratories (Kagoshima, Japan), respectively. Methanol and acetonitrile were of analytical grade for HPLC and other reagents used were of reagent grade.

### Preparation of calibration samples and quality control samples

A stock solution of lenvatinib was prepared by dissolving in methanol (3000 μg/mL). The stock solution was diluted with methanol to make working standard solutions of lenvatinib at the concentrations of 0.005, 0.01, 0.03, 1, 3, 10, 30, and 100 μg/mL. The stock solution was diluted with methanol to prepare solutions for preparing quality control (QC) for a reproducibility assessment at 0.005, 0.01, 0.3, 1, and 100 μg/mL, while those for stability assessments were 0.3, 30, and 2400 μg/mL. The stock solution of the IS was prepared in methanol (100 μg/mL) and was diluted by methanol to make a working solution at 0.5 μg/mL. The stock and working solutions for lenvatinib and the IS were refrigerated and used within the duration ensured in the validation study. The standard solutions were stable for 6 h at ambient temperature and for 28 days at 5 ± 4 °C.

Calibration samples and QC samples for the reproducibility assessment were prepared by spiking 0.05 mL of the working standard solutions to blank plasma (0.05 mL), and QC samples for the stability assessment were prepared by fortifying 0.05 mL of the solution to 1.5 mL of bank plasma. Lenvatinib concentrations in plasma were 0.005, 0.01, 0.03, 0.1, 0.3, 1, 3, 10, 30, and 100 μg/mL for the calibration samples, and were 0.005, 0.01, 1, and 100 μg/mL for the QC samples for reproducibility assessment. Lenvatinib concentrations of QC samples for the stability assessment were 0.01, 1, and 80 μg/mL.

### Extraction of lenvatinib

Methanol (50 μL) and the IS solution (0.5 μg/mL) were added to 50 μL of plasma samples. Then, 1 mL of 0.1 mol/L phosphate buffer (pH 8) and 4 mL of diethyl ether were added to these samples, and shaken for 10 min. The mixture was centrifuged (3000 rpm, 10 min, 4 °C) and the organic layer was transferred to a tube. Diethyl ether (4 mL) was added again to the aqueous layer, and shaken for 10 min. The mixture was centrifuged and the organic layer was transferred to a tube. 0.1 mol/L hydrochloric acid (200 μL) was added to the combined organic layer, shaken for 10 min, and the mixture was centrifuged. After removing the organic layer by aspiration followed by a stream of nitrogen gas to completely remove the dissolved diethyl ether in aqueous layer, a 55 μL aliquot of processed samples was injected into the HPLC system.

### Chromatographic conditions

The chromatographic condition for the determination of lenvatinib in plasma was consistent among the four different species. The HPLC system comprising of a separation module (model 2690) and a UV detector (model 2487) was used (Waters, Milford, MA). Lenvatinib and the IS were chromatographed on an analytical column, Mightysil RP-18 GP (4.6 mm inner diameter × 250 mm, 5 μm, Kanto chemical Co., Tokyo, Japan) at the column temperature of 40 °C. The mobile phase comprising of water with 50 mM sodium dodecyl sulfate (pH 2.5)/acetonitrile (55.5/45, v/v) was used at the flow rate of 1.2 mL/min. Lenvatinib was detected at the ultraviolet wavelength of 253 nm.

### Method validation

The developed method for the determination of lenvatinib concentrations in plasma was validated and the following validation parameters including linearity, selectivity, intra- and inter-batch reproducibility, extraction recovery, and stability were evaluated in accordance with the bioanalytical guidelines by United States Food and Drug Administration (US-FDA)^7^. The peak height ratios of lenvatinib to the IS of calibration samples were plotted against the corresponding nominal concentrations of lenvatinib. The calibration curve was prepared by the least square method with 1/(concentration)^2^ as the weighting factor. In the linearity assessment, accuracy of each calibration sample was determined to ensure that relative error (RE) at each concentration was within ± 15% (within ± 20% at the LLOQ). In the selectivity assessment, peak heights at the retention times of lenvatinib and the IS should be within 20% and 5% of those of the LLOQ for lenvatinib and the IS, respectively in blank plasma from six individuals. Intra-batch accuracy and precision were evaluated using QC samples at the four concentrations, LLOQ, low QC (LQC), mid QC (MQC), and ULOQ. Six replicates per concentration were assayed to calculate RE and relative standard deviation (RSD). Inter-batch accuracy and precision were evaluated by testing a single sample at each concentration across five batches (*n* = 5 in total). The acceptance criteria for RE and RSD in the intra- and inter-batch reproducibility should be within ± 15% and 15%, respectively, except at the LLOQ that was within ± 20% and 20%, respectively, was allowed. Extraction recoveries of lenvatinib and the IS from plasma samples of the four species were evaluated. The recoveries of lenvatinib at the low, mid, and high QC levels (triplicates per concentration), and that of the IS (0.5 μg/mL) were determined. Peak heights of lenvatinib after extraction from plasma samples were compared to those of the neat solution with the same concentrations. Stability of lenvatinib in plasma and processed samples was assessed at the low and high QC levels. Lenvatinib concentrations in samples after storage were compared to the theoretical concentrations and RE values were calculated. The RE values should be within ± 15% when considered stable. Freeze–thaw stability in plasma was evaluated up to three freeze–thaw cycles and frozen stability in plasma was evaluated at − 15 °C or below for 4 weeks (mice, rats, and dogs) or 6 weeks (monkeys). Stability in processed samples was assessed at 4 °C for 7 days. Stability of lenvatinib in the standard solutions was evaluated for the stock (100 μg/mL) and the working solution (5 ng/mL). The stability of the IS solution (0.5 μg/mL) was also assessed. The standard solutions were stored at 5 °C for 4 weeks or 6 h at ambient temperature. The peak height of the stored solutions was compared to that of the freshly prepared solution. The percentage of difference should be within ± 15% when considered stable.

### Pharmacokinetic studies

Animal care and experimental procedures were performed in the animal facility accredited by the Health Science Center for Accreditation of Laboratory Animal Care and Use of the Japan Health Sciences Foundation. All protocols were approved by the Institutional Animal Care and Use Committee at Eisai Co., Ltd. and Shin Nippon Biomedical Laboratories, Ltd. (Kagoshima, Japan), and carried out in accordance with the Animal Experimentation Regulations. The study is reported in accordance with ARRIVE guidelines.

#### PK study in mice

Female BALB/cAnNCrj-nu/nu mice were purchased from Charles River Laboratories (Kanagawa, Japan) and were acclimatized for 9 days prior to use in the PK study. At the dosing day, 7-week-old mice were used. E7080 was dissolved in 5% glucose injection to make a 0.3 mg/mL solution for intravenous administration and dissolved in water to make 0.3, 1, and 3 mg/mL solutions for oral administrations. Lenvatinib was intravenously administered at the dose of 3 mg/kg via the tail vein using a disposable syringe and oral administration was performed by gastric gavage at the doses of 3, 10, and 30 mg/kg at the dosing volume of 10 mL/kg (n = 3/time point/dose). Whole blood samples were drawn from the vena cava using heparinized syringes at 5, 15, and 30 min, and 1, 2, 4, 6, 8, 12, and 24 h postdose. Plasma samples were obtained by centrifugation and aliquots of plasma were transferred to a tube and stored below − 15 °C until assayed.

#### PK study in rats

Sprague–Dawley male rats were purchased from Charles River Japan, Inc., and were used for the PK study after 8 days of an acclimatization period. Rats were 8 weeks old when lenvatinib was dosed and the body weight range was 0.28–0.31 (kg). Rats were fasted a day before the PK study and feeding was resumed 4 h postdose. Dosing solution of lenvatinib used in the rat PK study was the same as that in the mice PK study, and the dose levels were 3 mg/kg for the intravenous administration via femoral vein, and 3, 10, and 30 mg/kg for the oral administration by gastric gavage (n = 4/dose). Approximately 0.25 mL of blood samples were obtained from the jugular vein using heparinized syringes at predose, and at 5, 15 and 30 min, and 1, 2, 4, 6, 8, 12 and 24 h postdose. Plasma sample preparation and sample storage were the same as those in the mice PK study.

#### PK study in dogs

Male beagle dogs purchased from Marshall Farm USA Inc. (NY, USA) were used for the PK study. Dogs were 16 months old when the PK study was conducted. The PK study was performed in a cross-over design with 8 days interval between the doses. Dogs were fasted a day before the PK study and feeding was resumed 4 h postdose. Dosing solution of lenvatinib used in the dog PK study was the same as that in the rodent PK studies. Lenvatinib was intravenously administered at the dose of 3 mg/kg via cephalic vein. Oral administration of lenvatinib was conducted at 3 mg/kg by using a tube with a disposable syringe, and then the dosing tube was given ca. 30 mL of water to flush the remaining dosing solution in the tube. The number of dogs at each dose was four. Approximately 0.5 mL of blood samples were serially obtained from the cephalic vein using a heparinized syringe at pre-dose, and at 5, 15 and 30 min, and 1, 2, 4, 6, 8, 12 and 24 h post-dose. Plasma sample preparation and sample storage were the same as those in the rodent PK studies.

#### PK study in monkeys

Male cynomolgus monkeys were supplied from Prestasi Fauna Nusantara Ltd. (Jakarta, Indonesia) and were housed at Shin Nippon Biomedical Laboratories, Ltd. The PK study was performed in a cross-over design with 7 days interval between the doses for monkeys aged 3–4 years old. Monkeys were fasted a day before the PK study and feeding was resumed 4 h postdose. Dosing solution of lenvatinib used was the same as that in the other animal PK studies. Lenvatinib was intravenously administered at the dose of 3 mg/kg via cephalic vein. In oral administration, lenvatinib at 3 mg/kg was dosed by using a nasogastric catheter, followed by giving 30 mL of water to flush the remaining dosing solution in the catheter. The number of monkeys at each dose was four. Approximately 0.5 mL of blood samples were serially collected from the femoral vein using a heparinized syringe at pre-dose, and at 5, 15 and 30 min, and 1, 2, 4, 6, 8, 12 and 24 h post-dose. Plasma sample preparation and sample storage were the same as those in the other PK studies.

#### PK analysis

PK parameters of lenvatinib were estimated by model independent analysis using a WinNonlin program (Version 4.1, Pharsight Corporation, Mountain View, CA). The time to reach the maximum plasma concentration (T_max_) and the maximum plasma concentration (C_max_) values were obtained directly from the plasma concentration data. Terminal half-life (T_1/2_) was calculated by ln2/*ke*, where *ke* is the slope of the regression line that fits the portion of the terminal phase (at least three time points) in the log-linear concentration–time curve. AUC_0–t_ and AUMC_0–t,_ the area under the concentration–time curves and the area under the first moment curve, from time = zero to time = t, respectively, were calculated using the lenvatinib concentrations in plasma by the linear/log trapezoidal method. AUC_0–inf_ was calculated by AUC_0–t_ + C_t_/*ke*, and AUMC_0–inf_ was AUMC_0–t_ + C_t_ × t/*ke* + C_t_/(*ke*)^2^, where C_t_ is the last measurable concentration at time = t. The plasma concentration at time zero for the intravenous dose was extrapolated, while zero was used for the oral doses. Mean residence time (MRT), total plasma clearance (CL_tot_) and the volume of distribution at steady state (V_dss_) were calculated by AUMC_0–inf_/AUC_0–inf_, Dose/AUC_0–inf_ and CL_tot_ × MRT, respectively. Oral bioavailability (F) was estimated by comparing dose-normalized AUC_0–inf_ after oral (AUC_0–inf.po_) and intravenous (AUC_0–inf.iv_) administration as follows: F (%) = (AUC_0–inf.po_/AUC_0–inf.iv_) × 100.

#### Allometric PK scaling

One of the typical approaches for predicting human PK from animal PK is empirical allometric scaling^6^. As the CL_tot_ of lenvatinib was relatively low and V_dss_ of lenvatinib was low to medium, thus it is theoretically preferable to correct by f_p_ for both parameters. The CL_tot_ divided by f_p_ (CL_tot_/f_p_) or V_dss_ divided by f_p_ (V_dss_/f_p_) were plotted against body weight (BW) by log–log scale, and a linear regression was performed to estimate a slope and intercept for linear regression. The BW used in the calculation was 0.02, 0.2, 10, 5, and 70 kg for mice, rats, dogs, monkeys, and humans, respectively. CL_tot_ and V_dss_ of lenvatinib in humans were predicted by using the regression line and the BW of humans. As no PK data of lenvatinib are available for CL_tot_ and V_dss_, which can be estimated only from intravenous administration PK study in humans, it is not possible to assess predictability of the allometric scaling for these two parameters of lenvatinib in humans. However, it is possible to predict exposure of lenvatinib after oral administration (AUC_oral_) in humans from the allometry scaling of hepatic intrinsic clearance (CL_int_). As lenvatinib is mainly cleared by the liver^8^, AUC_oral_ is estimated by Fa × Fg × Dose/(f_p_ × CL_int_)^9^, where Fa, Fg, f_p_, and CL_int_ are absorption at the gastrointestinal (GI) tract, metabolic availability at the GI, unbound fraction in plasma, and hepatic intrinsic clearance, respectively. The CL_int_ is thus estimated by Fa × Fg × Dose/(f_p_ × AUC_oral_) in all the animal species including humans just from PK data from oral administration. The allometric scaling approach was applied for CL_int_ in mice, rats, dogs, and monkeys. The logarithmic plot of CL_int_ and BW was performed for a linear regression and a regression line [log (CL_int_) = slope × log (BW) + intercept], which was derived by logarithmic transformation of the equation CL_int_ = a × BW^b^ was obtained. The CL_int_ predicted from the allometric scaling was compared to the observed CL_int_ after oral administration of lenvatinib at 9 mg in humans.

## Results and discussion

### Method validation

**Figure 1:**
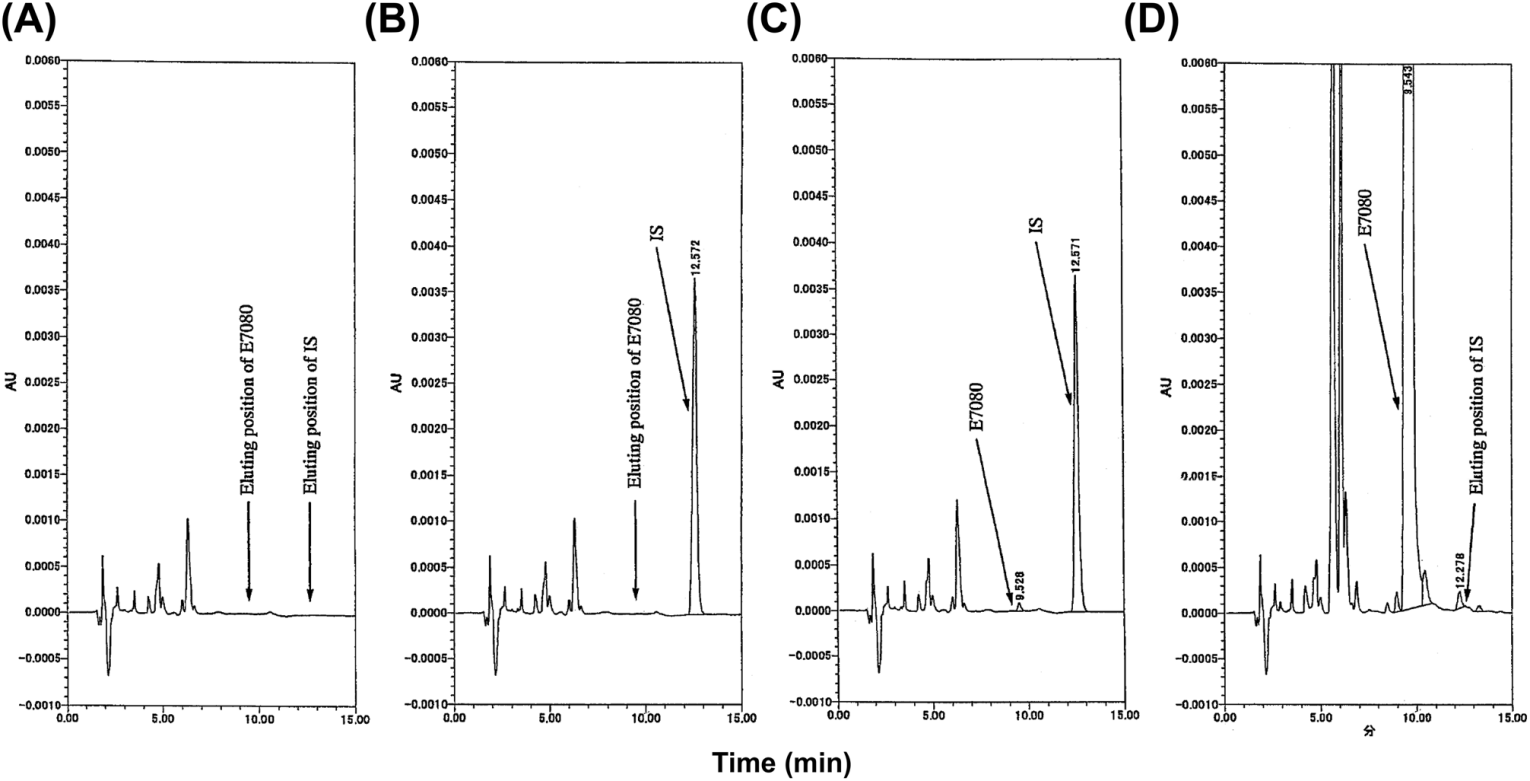
Typical chromatograms of lenvatinib in mice plasma. (**A**) blank sample, (**B**) zero sample in which only the internal standard was spiked, (**C**) low quality control (0.01 μg/mL) sample, and (**D**) upper limit of quantification (100 μg/mL) sample.

Method validation studies were performed in plasma from mice, rats, dogs, and monkeys with the same sample pretreatment and assay conditions. To achieve higher sensitivity by the traditional HPLC–UV method, liquid–liquid extraction rather than simple protein precipitation or solid phase extraction was selected for extraction of lenvatinib from plasma samples for removing unnecessary endogenous substances in plasma as much as possible. Validation parameters including selectivity, linearity, intra- and inter-batch reproducibility, extraction recovery, and stability were assessed in accordance with the bioanalytical guideline by US-FDA. In the selectivity test, no interfering peaks were detected at the retention times of lenvatinib and the IS in blank samples from six individuals in all the animal species (mice, rats, dogs, and monkeys). Figure 1 represented typical chromatograms of a blank sample (Fig. 1A), a zero sample in which only the IS was spiked (Fig. 1B), the LQC sample (Fig. 1C), and the ULOQ sample (Fig. 1D) in mice. Figure 1 showed chromatograms of mice plasma as a typical example, but similar chromatograms with no interferences were observed in the other three animal species. The retention times of lenvatinib and the IS were similar between the spiked LLOQ sample and the post-dose sample. No interfering peaks were observed at the retention time of lenvatinib in blank and zero samples, and no peaks were detected at the retention time of the IS in blank samples, indicating that selectivity was ensured in the assay. Results in the linearity assessment showed that RE values were within ± 15% at all the tested concentrations of calibration samples covering the 20,000-fold concentration range (0.005–100 μg/mL) and the correlation coefficient was 0.999 or higher, in all the tested species, ensuring the linearity of the assay. Equations of linear regression were y = 2.08x + 0.000940, y = 2.14x + 0.00499, y = 2.02x − 0.000464, y = 2.19x + 0.00220, in mice, rats, dogs, and monkeys, respectively. The linearity was evaluated across six assay batches and all the batches met the acceptance criteria.

Results of the intra- and inter-batch reproducibility tests across the four species are represented in Tables 1 and 2, respectively. In the intra-batch test, the RE and RSD values were within ± 15% and 15%, respectively, at the tested four QC levels (LLOQ, LQC, MQC, and ULOQ). The RE and RSD in the inter-batch test were also within ± 15% and 15%, respectively. These results indicate that the assay is reproducible. The peak height of lenvatinib at the LLOQ was at least five times of that in blank samples, showing sufficient signal-to-noise ratio at the LLOQ. The acceptable RE and RSD along with sufficient signal-to noise ratio set the LLOQ at 0.005 μg/mL.

**Table 1:** Intra-batch accuracy and precision of lenvatinib assay in plasma.

QC samples	Concentration (μg/mL)	Accuracy (% RE)	Precision (% RSD)
Mice			
LLOQ	0.005	2.0	5.9
LQC	0.01	8.0	1.9
MQC	1	0.4	0.9
ULOQ	100	− 2.3	1.7
Rats			
LLOQ	0.005	− 6.0	4.3
LQC	0.01	− 2.0	4.1
MQC	1	1.9	1.6
ULOQ	100	− 3.3	1.0
Dogs			
LLOQ	0.005	10.0	5.5
LQC	0.01	6.0	3.8
MQC	1	1.4	1.0
ULOQ	100	− 4.5	1.7
Monkeys			
LLOQ	0.005	− 15.0	10.4
LQC	0.01	− 5.0	6.4
MQC	1	4.1	1.6
ULOQ	100	− 0.7	2.0

*LLOQ* lower limit of quantification, *LQC* low QC, *MQC* mid QC, *ULOQ* upper limit of quantification, *QC* quality control, *RE* relative error, *RSD* relative standard deviation.

Six replicates per concentration (LLOQ, LQC, MQC, and ULOQ) in a batch were assayed to estimate accuracy and precision.

**Table 2:** Inter-batch accuracy and precision of lenvatinib assay in plasma.

QC samples	Concentration (ng/mL)	Accuracy (% RE)	Precision (% RSD)
Mice			
LLOQ	0.005	6.0	3.8
LQC	0.01	6.0	3.8
MQC	1	0.5	0.6
ULOQ	100	− 0.8	1.5
Rats			
LLOQ	0.005	6.0	3.8
LQC	0.01	3.0	3.9
MQC	1	2.4	1.6
ULOQ	100	− 3.1	3.9
Dogs			
LLOQ	0.005	8.0	3.7
LQC	0.01	5.0	3.8
MQC	1	4.0	3.1
ULOQ	100	− 2.9	3.0
Monkeys			
LLOQ	0.005	12.4	8.5
LQC	0.01	5.2	5.6
MQC	1	3.4	1.1
ULOQ	100	− 2.5	1.6

*LLOQ* lower limit of quantification, *LQC* low QC, *MQC* mid QC, *ULOQ* upper limit of quantification, *QC* quality control, *RE* relative error, *RSD* relative standard deviation.

One QC sample at each concentration (LLOQ, LQC, MQC, and ULOQ) was assayed across 5 days and accuracy and precision were calculated (*n* = 5 per concentration).

Extraction recoveries of lenvatinib and the IS were determined at the three concentration levels (LQC, MQC, and HQC). The recovery of lenvatinib was high (> 77%) in all the tested species and was consistent, regardless of the tested concentrations (Table 3). The recovery of the IS was similar to that of lenvatinib.

**Table 3:** Extraction recovery of lenvatinib and the internal standard from plasma.

Drugs	QC samples	Recovery (%)			
		Mice	Rats	Dogs	Monkeys
Lenvatinib	LQC	77.1 ± 1.3	81.4 ± 2.2	77.8 ± 2.6	92.9 ± 9.3
	MQC	83.3 ± 0.4	79.5 ± 0.4	84.5 ± 0.9	84.6 ± 1.2
	HQC	84.2 ± 1.2	82.4 ± 1.0	82.9 ± 1.5	84.7 ± 1.4
IS		82.3 ± 1.1	79.4 ± 1.1	81.7 ± 1.0	88.4 ± 1.2

*HQC* high QC, *IS* internal standard, *LQC* low QC, *MQC* mid QC, *QC* quality control.

Triplicates per concentration were assayed and mean ± standard deviation of extraction recovery was represented.

Stability of lenvatinib was assessed at the three concentrations using LQC, MQC, and HQC samples. The percentage bias of the samples from the theoretical values were within ± 15% at the three concentration levels in all the tested animal species (Table 4). These findings indicate that the freeze/thaw stability in plasma after three cycles, frozen stability in plasma for at least 4 weeks, bench-top stability at 37 °C for 96 h, and processed sample stability at 4 °C for 7 days were ensured in mice, rats, dogs, and monkeys.

**Table 4:** Stability assessment of lenvatinib.

Stability	Condition	QC samples	Mice	Rats	Dogs	Monkeys
Freeze/thaw	3 cycles	LQC	− 2.9	− 7.9	− 2.9	− 5.0
		MQC	− 1.7	3.6	1.5	2.3
		HQC	0.8	− 3.5	0.6	1.6
Frozen	Below − 15 °C, 4W or 6W	LQC	0.0	1.0	− 6.7	2.0
		MQC	1.0	− 1.5	− 1.7	4.2
		HQC	− 0.1	− 3.5	3.1	7.0
Bench-top	37 °C, 96 h	LQC	− 8.8	− 4.0	− 12.9	− 7.0
		MQC	− 8.2	− 8.7	− 12.9	− 6.0
		HQC	− 6.5	− 4.9	− 12.4	− 7.4
Processed	4 °C, 7 days	LQC	− 2.0	− 1.9	− 2.8	− 3.7
		MQC	− 0.1	0.2	− 0.3	− 0.3
		HQC	0.3	0.3	− 0.1	− 0.2

*HQC* high QC, *LQC* low QC, *MQC* mid QC, *QC* quality control.

Data represent the mean percentage bias from the theoretical values (n = 3 at each concentration). Duration for the frozen stability was 4 weeks for mice, rats, and dogs, while 6 weeks for monkeys.

### Pharmacokinetic studies

**Figure 2:**
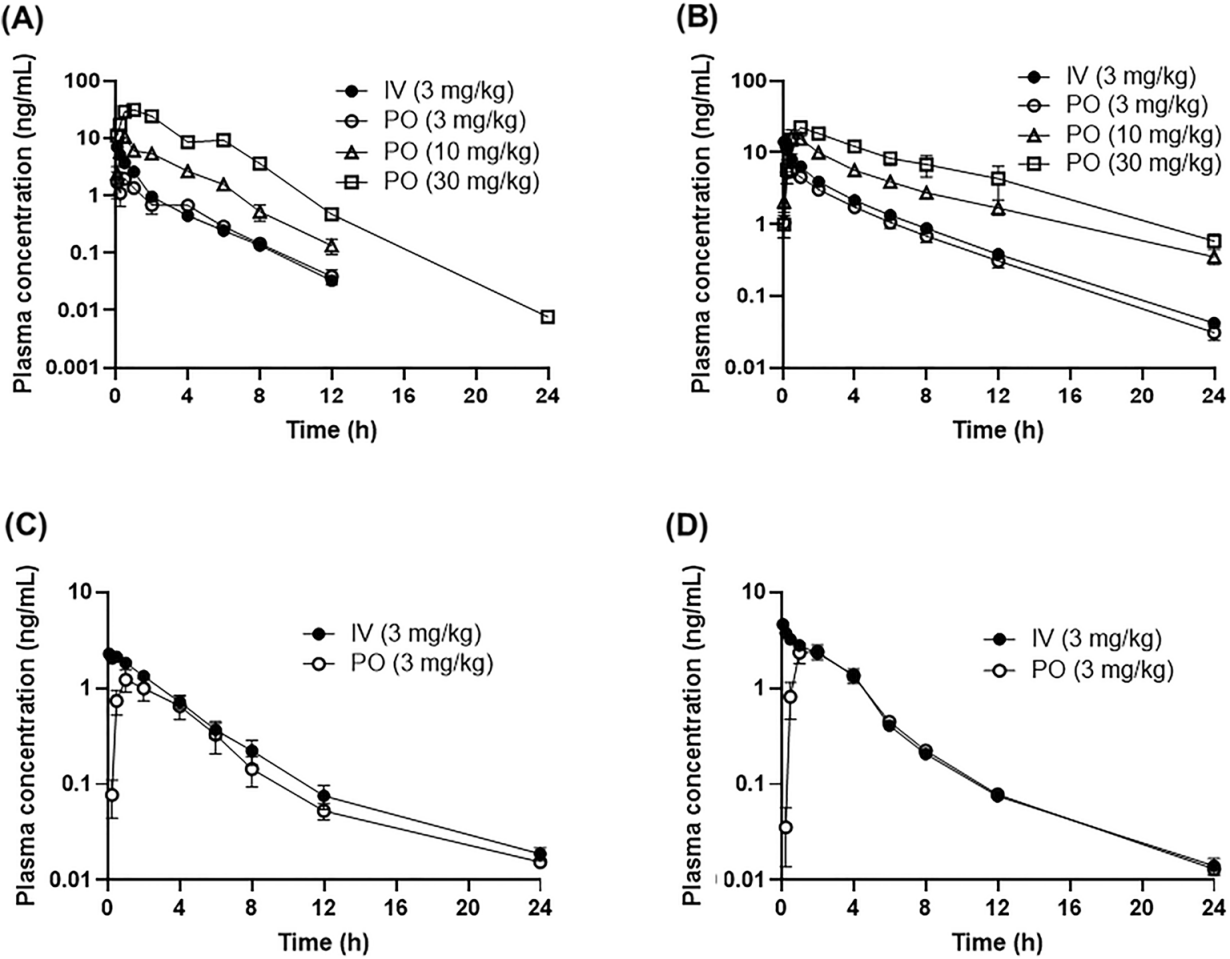
Plasma concentration time profiles of lenvatinib in mice (**A**), rats (**B**), dogs (**C**), and monkeys (**D**). Data represent the mean for mice, while the mean ± standard deviation for rats, dogs, and monkeys. Lenvatinib was dosed orally (3, 10, and 30 mg/kg) or intravenously (3 mg/kg) to mice (*n* = 3), orally (3, 10, and 30 mg/kg) or intravenously (3 mg/kg) to rats (*n* = 4), orally (3 mg/kg) or intravenously (3 mg/kg) to dogs (*n* = 4), and orally (3 mg/kg) or intravenously (3 mg/kg) to monkeys (*n* = 4). Blood samples were obtained and prepared plasma samples were assayed by the validated methods for the determination of lenvatinib concentrations in plasma.

Nonclinical PK profiles of lenvatinib were investigated in mice, rats, dogs, and monkeys. Lenvatinib was intravenously administered at one dose level (3 mg/kg) in the four animal species and orally dosed at the three dose levels in mice and rats (3, 10, and 30 mg/kg), while at one level (3 mg/kg) in dogs and monkeys. Obtained plasma samples were assayed for the determination of lenvatinib concentrations by the validated method. PK profiles of lenvatinib after intravenous and oral doses are represented in Fig. 2 and PK parameters of lenvatinib in the four animals are represented in Tables 5 and 6 for intravenous and oral doses, respectively.

**Table 5:** Pharmacokinetic parameters of lenvatinib after intravenous administration.

Parameters	Unit	Species			
		Mice	Rats	Dogs	Monkeys
Dose	mg/kg	3	3	3	3
T_1/2_	h	2.05	3.65 ± 0.09	5.27 ± 0.83	4.28 ± 0.34
AUC_0-inf_	μg × h/mL	8.69	30.1 ± 1.33	8.42 ± 0.927	12.9 ± 1.06
CL_tot_	mL/h/kg	345	100 ± 4.3	368 ± 36.3	238 ± 21.1
V_dss_	mL/kg	714	392 ± 9.9	1610 ± 249	794 ± 88.2
C_0_	μg/mL	8.29	16.0 ± 0.416	2.44 ± 0.303	5.17 ± 0.949
MRT	h	2.07	3.93 ± 0.19	4.34 ± 0.36	3.32 ± 0.15

*AUC*_*0-inf*_ area under the concentration time profile from time zero to infinity, *C*_*0*_ extrapolated maximum concentration at time = zero, *CL*_*tot*_ total clearance, *MRT* mean residence time, *T*_*1/2*_ elimination half-life, *V*_*dss*_ volume of distribution at the steady-state.

Data represent the mean of three mice, while the mean ± standard deviation of four rats, four dogs, and four monkeys.

**Table 6:** Pharmacokinetic parameters of lenvatinib after oral administration.

Parameters	Unit	Species							
		Mice			Rats			Dogs	Monkeys
Dose	mg/kg	3	10	30	3	10	30	3	3
T_1/2_	h	2.09	1.74	1.85	3.61 ± 0.21	5.27 ± 0.33	4.95 ± 0.77	4.76 ± 0.94	4.07 ± 0.29
AUC_0-inf_	μg × h/mL	5.60	27.7	118	20.7 ± 3.12	78.3 ± 6.58	146 ± 18.5	5.48 ± 1.37	10.3 ± 1.51
C_max_	μg/mL	1.97	10.5	31.3	6.17 ± 1.37	16.6 ± 1.24	23.2 ± 4.77	1.27 ± 0.302	2.50 ± 0.450
T_max_	h	0.5	0.5	1	0.5 (0.25–0.5)	0.5 (0.25–1)	1 (1–2)	2 (0.5–2)	2 (1–2)
MRT	h	3.28	3.01	3.29	4.46 ± 0.16	6.31 ± 0.53	6.86 ± 1.02	4.81 ± 0.25	4.23 ± 0.31
F	%	64.4	N.C	N.C	68.7	N.C	N.C	70.4 ± 23.1	78.4 ± 5.4

*AUC*_*0-inf*_ area under the concentration time profile from time zero to infinity, *C*_*max*_ maximum concentration, *F* bioavailability, *MRT* mean residence time, *NC* not calculated, *T*_*1/2*_ elimination half-life, *T*_*max*_ time to reach maximum concentration.

Data represent the mean of three mice, while the mean ± standard deviation of four rats, four dogs, and four monkeys, while median and data range in parenthesis are represented for T_max_.

In mice after intravenous administration, lenvatinib eliminated rapidly with T_1/2_ of 2.05 h (Fig. 2A). The CL_tot_ and V_dss_ were 345 mL/h/kg and 714 mL/kg, respectively, indicating low clearance and low volume of distribution. After oral administration to mice, lenvatinib reached the C_max_ at 0.5–1 h and eliminated with T_1/2_ of 1.74–2.09 h at 3, 10, and 30 mg/kg, which indicated rapid absorption after the oral doses. The C_max_ values were 1.97, 10.5, and 31.3 μg/mL, respectively, and AUC_0-inf_ values were 5.60, 27.7, and 118 μg × h/mL, respectively, at 3, 10, and 30 mg/kg. The C_max_ and AUC_0-inf_ increased almost dose-proportionally, indicating that the systemic exposure of lenvatinib was almost linear from 3 to 30 mg/kg after the oral doses. The oral bioavailability in mice at 3 mg/kg was 64.4%.

In rats, after intravenous administration, lenvatinib eliminated relatively rapidly with T_1/2_ of 3.65 h (Fig. 2B). The CL_tot_ and V_dss_ were 100 mL/h/kg and 392 mL/kg, respectively, indicating low clearance and low volume of distribution. After oral administration to rats, lenvatinib reached the C_max_ at 0.5–1 h and eliminated with T_1/2_ of 3.61–5.27 h at 3, 10, and 30 mg/kg, respectively. The C_max_ values were 6.17, 16.6, and 23.2 μg/mL, respectively, and AUC_0-inf_ values were 20.7, 78.3, and 146 μg × h/mL, respectively, at 3, 10, and 30 mg/kg. The C_max_ and AUC_0-inf_ increased dose-proportionally, indicating that the systemic exposure of lenvatinib after oral doses was almost linear up to 30 mg/kg. The oral bioavailability in rats at 3 mg/kg was 68.7%. The rapid absorption in the oral PK and low CL_tot_ and V_dss_ in the intravenous PK in rats showed similar PK characteristics of lenvatinib in the two rodent species (mice and rats). The CL_tot_ of lenvatinib in rats (100 mL/h/kg) was lower than the other three species (238–368 mL/h/kg). As the CL_tot_ of lenvatinib in the four animal species was relatively lower, the CL_tot_ was governed by plasma unbound fraction and intrinsic metabolic clearance (CL_tot_ = f_p_ × CL_int_). Although the intrinsic clearance of lenvatinib was not assessed, the unbound fraction in rats was lower than the other species from the findings. The in vitro plasma protein binding of lenvatinib at 0.3 μg/mL was 96.9%, 98.1%, 91.6%, and 96.1% in mice, rats, dogs, and monkeys, respectively. It is thus speculated that relatively high plasma protein binding of lenvatinib in rats may lead to higher exposure levels of lenvatinib in rats compared to other animal species.

In dogs, after intravenous administration, lenvatinib eliminated with T_1/2_ of 5.27 h (Fig. 2C). The CL_tot_ and V_dss_ were 368 mL/h/kg and 1610 mL/kg, respectively, indicating low clearance and low to mid volume of distribution. After oral administration to dogs, lenvatinib reached the C_max_ at 2 h and eliminated with T_1/2_ of 4.76 h at 3 mg/kg. The C_max_ and AUC_0-inf_ values were 1.27 μg/mL and 5.48 μg × h/mL at 3 mg/kg. The oral bioavailability in dogs at 3 mg/kg was 70.4%. When PK parameters in dogs were compared to those in rodents, dogs showed similar CL_tot_ and oral exposure at 3 mg/kg to those in mice.

In monkeys, after intravenous administration, lenvatinib eliminated with T_1/2_ of 4.28 h (Fig. 2D). The CL_tot_ and V_dss_ were 238 mL/h/kg and 794 mL/kg, respectively, indicating low clearance and low volume of distribution. After oral administration to monkeys, lenvatinib reached the C_max_ at 2 h and eliminated with T_1/2_ of 4.07 h at 3 mg/kg. The C_max_ and AUC_0-inf_ values were 2.50 μg/mL and 10.3 μg × h/mL at 3 mg/kg. The oral bioavailability in monkeys at 3 mg/kg was 78.4%. The exposure after the oral dose in monkeys was approximately twofold higher than that in dogs. In the PK studies in dogs and monkeys, only one oral dose was tested. However, in separate toxicokinetic studies in dogs and monkeys, three dose levels up to 30 mg/kg oral doses were tested. PK profiles of lenvatinib were almost linear up to 30 mg/kg oral doses in both dogs and monkeys. Thus, only one dose at 3 mg/kg was selected in the PK studies in dogs and monkeys since the PK characteristics of lenvatinib is evident at one dose under linear PK profiles. The PK parameters in the four animal species demonstrated that CL_tot_ and V_dss_ values were relatively low and oral bioavailability was high ranging from 64.4 to 78.4%, which is similar among the tested doses. These findings imply that PK characteristics of lenvatinib is similar between the tested four animals species.

**Figure 3:**
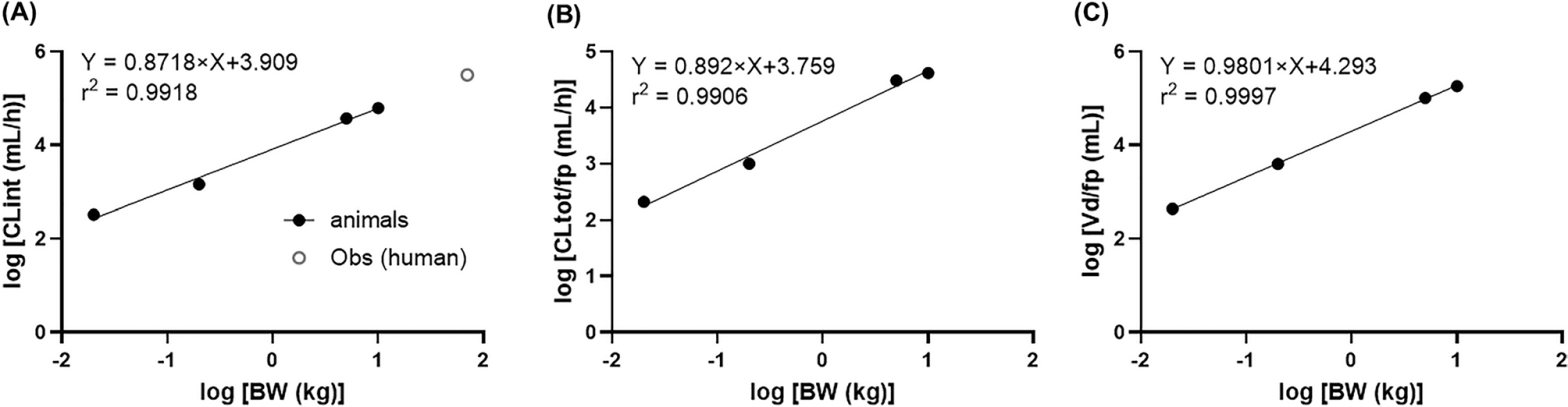
Allometric scaling of intrinsic clearance, total clearance, and volume of distribution from nonclinical study. The intrinsic clearance (CL_int_) (**A**), total clearance (CL_tot_) divided by plasma unbound fraction (f_p_) (**B**), and volume of distribution (V_dss_) divided by f_p_ (**C**), from nonclinical pharmacokinetic (PK) study were plotted against body weight (BW). The BW was assumed 0.02, 0.2, 10, and 5 kg for mice, rats, dogs, and monkeys, respectively. A linear regression of the relationship between log (BW) and log (PK parameters) was obtained. The CL_int_ in humans was calculated to be 4508 mL/h/kg from AUC_oral_ at 9 mg oral dose of lenvatinib in a phase-1 study and the average plasma unbound fraction (0.0172).

We analyzed, retrospectively, whether CL_tot_ of lenvatinib in humans can be predicted from that in nonclinical animals using the empirical cross-species allometric scaling approach. The CL_int_ of lenvatinib was estimated to be 325, 1440, 61,500, and 37,063 (mL/h) in mice, rats, dogs, and monkeys, respectively. This is based on the assumption that absorption and metabolic availability of lenvatinib at the GI is complete (*i.e.,* both Fa and Fg are unity) and on the finding that the average f_p_ values of lenvatinib at 0.3–30 μg/mL were 0.033, 0.020, 0.089, and 0.0394, respectively, in mice, rats, dogs, and monkeys. It is possible that availability in the intestine is not complete in animal species and humans. However, availability in the intestine is considered to be similar and relatively high (> 0.7) when availability of lenvatinib in the intestine is calculated from CL_tot_ after an intravenous dose, blood-to-plasma partition, hepatic blood flow, and bioavailability after an oral dose on the assumption that CL_tot_, after an intravenous dose, was the same as the hepatic clearance. The linear regression of the correlation between log (CL_int_, mL/h) and log (BW, kg) resulted in the equation log (CL_int_) = 3.909 + 0.8718 × log (BW) by cross-species allometric scaling (Fig. 3A). CL_int_ of lenvatinib in humans was predicted to be 4704 mL/h/kg, which was very close to the observed CL_int_ of lenvatinib in a phase-1 clinical trial of lenvatinib (4508 mL/h/kg, calculated from AUC_oral_ at 9 mg^10^). The slope in the equation was in good agreement with the expected values^6^.

The V_dss_ or CL_tot_ of lenvatinib after intravenous administration in the four nonclinical animal species divided by f_p_ were also plotted against BW. The correlation coefficient of the plot was remarkably increased by correction with f_p_ for both CL_tot_ and V_dss_ (data not shown). The obtained regression equations were log (CL_tot_/f_p_) = 3.759 + 0.892 × log (BW) and log (V_dss_/f_p_) = 4.293 + 0.9801 × log (BW), in which the slopes in both equations were also within the reasonable range (Fig. 3B, C). As no intravenous PK data of lenvatinib are available in humans, it is not possible to compare directly between observed and predicted values for CL_tot_ and V_dss_. If we assume that the bioavailability of lenvatinib in humans is similar to that in the four animal species given that similar bioavailability was noted in the four animal species, and an average bioavailability of lenvatinib in the four species (0.70) was used, it is possible to predict CL_tot_ and V_dss_ values in humans. However, a study by Mahmood suggested that it is sometimes erratic to assume that bioavailability in humans is like that in animals^6^, and thus CL_tot_ or V_dss_ of lenvatinib in humans were not predicted in this study.

Cross-species pharmacokinetics of other tyrosine kinase inhibitors have been reported for anlotinib^11^, nilotinib^12^, and pyrotinib^13^. Preclinical pharmacokinetics of nilotinib were reported in mice, dogs, and monkeys. The CL_tot_ values of nilotinib were 131, 190, and 639 mL/h/kg in mice, dogs, and monkeys, respectively, and the V_dss_ values were 290, 1157, and 5737 mL/kg, respectively. The CL_tot_ was relatively low in all the three species and V_dss_ was species-dependent: low in mice and high in monkeys. Oral bioavailability of nilotinib was 50, 24, and 16% in mice, dogs, and monkeys, respectively, indicating species-dependent PK of nilotinib. Pharmacokinetic parameters of anlotinib after intravenous administration to rats and dogs showed high CL_tot_ and V_dss_. Similar parameters were reported between male and female animals and thus pharmacokinetic parameters in male rats are listed here. The CL_tot_ in rats (6.34 L/h/kg) was higher than that in dogs (0.38 L/h/kg) and V_dss_ in rats was also higher than that in dogs (25.4 vs 6.8 L/kg). Bioavailability of anlotinib in rats ranged from 27.5 to 36.6% in rats and from 47.6 to 53.7% in dogs, which showed linear pharmacokinetics after oral doses in both animals. Nonclinical PK of pyrotinib was characterized in mice, rats, and dogs after intravenous and oral administration. The CL_tot_ of pyrotinib was 37.2, 3.5, and 28 (mL/min/kg), respectively, and V_dss_ was 5027, 1350, and 8157 (mL/kg), respectively, in mice, rats, and dogs. These parameters suggested that pyrotinib showed low clearance and high volume of distribution in the three tested species. The F was 20.6, 43.5, and 20.0% in mice, rats, and dogs, respectively, indicating that PK of pyrotinib is similar among the three species.

Since the PK parameters of the three tyrosine kinase inhibitors in humans after intravenous administration have not reported to the best of our knowledge, it is not possible to compare characteristics of CL_tot_ or V_dss_ between nonclinical animals and humans. However, it is possible to predict systemic exposure in humans after oral administration using the approach we proposed in this study. In vitro–in vivo correlation using human derived matrices (e.g., liver microsomes or hepatocytes) has been another option for predicting human PK, however, allometric scaling is still a useful approach for human PK prediction.

## Conclusion

A simple HPLC method with 20,000-fold concentration range was established and applied to PK studies of lenvatinib in mice, rats, dogs, and monkeys. The validation parameters were within the acceptance criteria and thus the assay was reproducible. In all the four animal species, lenvatinib showed relatively low clearance and low volume of distribution after intravenous administration and showed rapid and high absorption with high oral availability after oral dose. An empirical allometric scaling approach using PK data in nonclinical animals accurately predicted PK of lenvatinib in humans after oral dose.

## Data Availability

The data generated in this study are available from the corresponding author on reasonable request.

## References

[1] Matsui J, Yamamoto Y, Funahashi Y, Tsuruoka A, Watanabe T, Wakabayashi T, Uenaka T, Asada M (2008). E7080, a novel inhibitor that targets multiple kinases, has potent antitumor activities against stem cell factor producing human small cell lung cancer H146, based on angiogenesis inhibition. Int. J. Cancer.

[2] Matsui J, Funahashi Y, Uenaka T, Watanabe T, Tsuruoka A, Asada M (2008). Multi-kinase inhibitor E7080 suppresses lymph node and lung metastases of human mammary breast tumor MDA-MB-231 via inhibition of vascular endothelial growth factor-receptor (VEGF-R) 2 and VEGF-R3 kinase. Clin. Cancer Res..

[3] Okamoto K, Kodama K, Takase K, Sugi NH, Yamamoto Y, Iwata M, Tsuruoka A (2013). Antitumor activities of the targeted multi-tyrosine kinase inhibitor lenvatinib (E7080) against RET gene fusion-driven tumor models. Cancer Lett..

[4] Mano Y (2018). Method validation studies and an inter-laboratory cross validation study of lenvatinib assay in human plasma using LC-MS/MS. Pract. Lab. Med..

[5] Zanchetta M, Iacuzzi V, Posocco B, Bortolin G, Poetto AS, Orleni M, Canil G, Guardascione M, Foltran L, Fanotto V, Puglisi F, Gagno S, Toffoli GA (2021). Rapid, simple and sensitive LC-MS/MS method for lenvatinib quantification in human plasma for therapeutic drug monitoring. PLoS One.

[6] Mahmood I (2009). Pharmacokinetic allometric scaling of antibodies: Application to the first-in-human dose estimation. J. Pharm. Sci..

[7] US Department of Health and Human Services, F. D. A., Center for Drug Evaluation and Research (CDER), Center for Veterinary Medicine (CVM). Guidance for Industry: Bioanalytical Method Validation. https://www.fda.gov/downloads/drugs/guidancees/ucm070107.pdf (2001).

[8] Hussein Z, Mizuo H, Hayato S, Namiki M, Shumaker R (2017). Clinical pharmacokinetic and pharmacodynamic profile of lenvatinib, an orally active, small-molecule, multitargeted tyrosine kinase inhibitor. Eur. J. Drug Metab. Pharmacokinet..

[9] Benet LZ, Hoener BA (2002). Changes in plasma protein binding have little clinical relevance. Clin. Pharmacol. Ther..

[10] Yamada K, Yamamoto N, Yamada Y, Nokihara H, Fujiwara Y, Hirata T, Koizumi F, Nishio K, Koyama N, Tamura T (2011). Phase I dose-escalation study and biomarker analysis of E7080 in patients with advanced solid tumors. Clin. Cancer Res..

[11] Zhong CC, Chen F, Yang JL, Jia WW, Li L, Cheng C, Du FF, Zhang SP, Xie CY, Zhang NT, Olaleye OE, Wang FQ, Xu F, Lou LG, Chen DY, Niu W, Li C (2018). Pharmacokinetics and disposition of anlotinib, an oral tyrosine kinase inhibitor, in experimental animal species. Acta Pharmacol. Sin..

[12] Ananthula HK, Parker S, Touchette E, Buller RM, Patel G, Kalman D, Salzer JS, Gallardo-Romero N, Olson V, Damon IK, Moir-Savitz T, Sallans L, Werner MH, Sherwin CM, Desai PB (2018). Preclinical pharmacokinetic evaluation to facilitate repurposing of tyrosine kinase inhibitors nilotinib and imatinib as antiviral agents. BMC Pharmacol. Toxicol..

[13] Li X, Yang C, Wan H, Zhang G, Feng J, Zhang L, Chen X, Zhong D, Lou L, Tao W, Zhang L (2017). Discovery and development of pyrotinib: A novel irreversible EGFR/HER2 dual tyrosine kinase inhibitor with favorable safety profiles for the treatment of breast cancer. Eur. J. Pharm. Sci..

